# Palmar Contracture

**Published:** 2013-01-18

**Authors:** Keith E. Follmar, Scott D. Lifchez

**Affiliations:** Department of Plastic and Reconstructive Surgery, The Johns Hopkins University, Baltimore, MD

**Figure:**
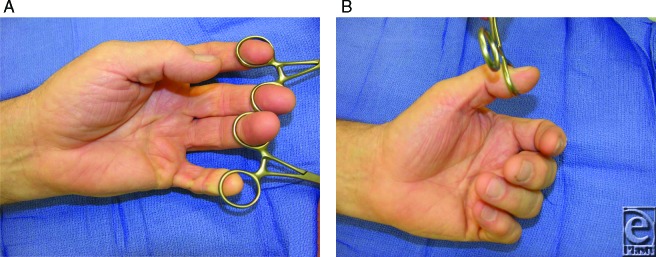


## DESCRIPTION

A 71-year-old right-hand-dominant retired man complains of inability to straighten the thumb, middle finger, and small finger of his left hand. This symptom has been progressively worsening over the past 2 or 3 years. On examination, the 3 affected digits cannot be fully extended, either passively or actively. There is a thick band of fibrous tissue tethering each digit in its contracted posture. The small finger has contracture at the metacarpophalangeal (MP), proximal interphalangeal (PIP), and distal interphalangeal (DIP) joints. The middle finger contracture is principally involving the MP joint. The thumb contracture involves the MP and IP joints.

## QUESTIONS

1. **What is the correct diagnosis?**
2. **What are the anatomic structures involved in this disease process?**
3. **What are the indications for therapeutic intervention?**
4. **What are the therapeutic options to treat this disease?**
5. **What are the surgical hazards to avoid while treating this disease surgically?**

## DISCUSSION

This patient's diagnosis is Dupuytren's contractures of the hand. Dupuytren's disease is a benign fibroproliferative process involving the palmar fascia, which results in contracture of the fingers.^1^ The small and ring fingers are the most commonly affected, and involvement of the middle finger is commonly seen as well. Involvement of the index finger and thumb (as in this patient) is very uncommon. Progression of the disease is generally slow (over years to decades) and typically painless. The condition has been associated with northern European ancestry, family history, alcoholism, epilepsy, chronic pulmonary disease, and diabetes.

The anatomic structures that can be involved with Dupuytren's disease include the pretendinous bands, the spiral band, the central band, the lateral digital sheet, Grayson's ligament, the natatory ligament, and the Septae of Legueu and Juvara.^2^ When the disease involves the pretendinous band, the spiral band, the lateral digital sheet, and Grayson's ligament, the resulting cord spirals around the digital neurovascular bundle. As this cord contracts, it becomes straight and distorts the course of the digital neurovascular bundle, such that the neurovascular bundle becomes spiraled around the cord.^3^ In the case of the small finger, the ulnar-sided cord can originate from the musculotendinous junction of the abductor digiti minimi muscle.

Early Dupuytren's disease presents with palmar nodules, which are typically asymptomatic. As cord formation progresses, the MP joint is typically the first to become contracted, followed by the PIP. Involvement of the DIP joint, as in our patient here, is an uncommon finding. Indications for intervention include greater than 30° of contracture of the MP joint, or any contracture of the PIP or DIP joint. Even with formal release of the joint, any contracture of the PIP joint commonly does not fully respond to intervention or recurs shortly thereafter. Indications of surgery can be adjusted on the basis of patients' symptoms and other medical conditions.

**Figure 1:**
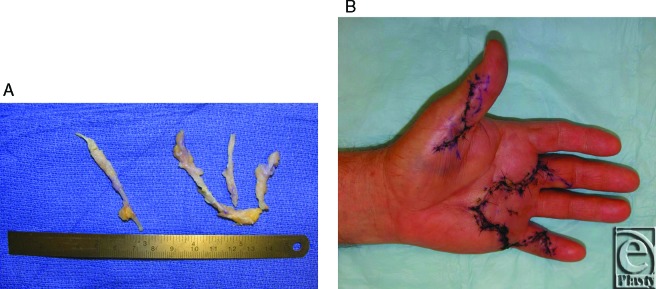
Dense cords of connective tissue (a) were excised from each of the affected digits through Brunner incisons (b). Great care was taken to not injure the digital neurovascular bundles.

Therapeutic options include collagenase injection followed by manipulation under local anesthesia,^4^ needle fasciotomy,^5^ limited fasciectomy, and complete palmar fasciectomy. Collagenase injection involves injecting Clostridial collagenase into the area of the pathologic cord. After allowing 24 to 48 hours for the cord to be enzymatically weakened, the cord is mechanically disrupted by bluntly manipulating the affected fingers under anesthesia. Needle fasciotomy is performed by sharply lacerating the palmar cord with a 16-gauze through a percutaneous approach while bluntly manipulating the affected fingers to hold tension on the cord. Palmar fasciectomy is performed by directly excising the affected fascia through an open approach with broad exposure (Fig 1). Limited fasciectomy also involves direct excision of the affected cord, but typically through a more limited incision.

**Figure 2:**
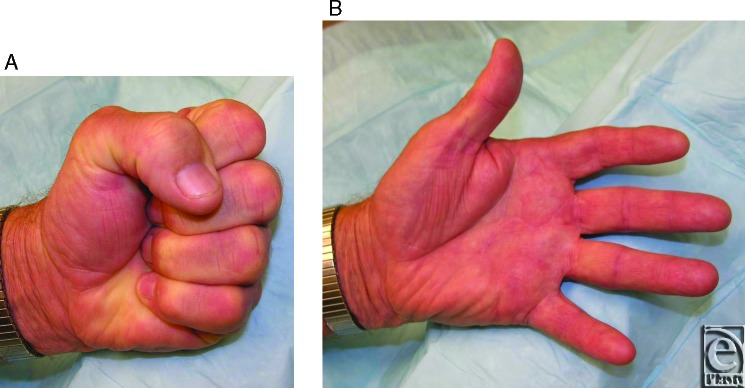
At postoperative day 207, the patient has complete flexion (a), and nearly-complete extension of all digits (b).

Complete fasciectomy can be performed through Brunner incisions, Y-to-V incisions, midlateral incisions, or longitudinal volar incision with Z-plasty tissue rearrangement at the time of closure.^6^ The most common complication is recurrence of contracture, which happens to at least a small extent in most cases (Fig 2). MP joint contractures are typically more responsive to therapy than are PIP joint contractures. Other complications include skin loss, infection, damage to digital nerves and arteries, loss of full flexion, and complex regional pain syndrome.^7^

## References

[1] Saar JD, Grothaus PC (2000). Dupuytren's disease: an overview. Plast Reconstr Surg.

[2] McFarlane RM (1974). Patterns of the diseased fascia in the fingers in Dupuytren's contracture: displacement of the neurovascular bundle. Plast Reconstr Surg.

[3] Hettiaratchy S, Tonkin MA, Edmunds IA (2010). Spiralling of the neurovascular bundle in Dupuytren's disease. J Hand Surg Eur Vol.

[4] Hurst LC, Badalamente MA, Hentz VR (2009). Injectable collagenase clostridium histolyticum for Dupuytren's contracture. N Engl J Med.

[5] Foucher G, Medina J, Navarro R (2003). Percutaneous needle aponeurotomy: complications and results. J Hand Surg Br.

[6] Citron ND, Nunez V (2005). Recurrence after surgery for Dupuytren's disease: a randomized trial of two skin incisions. J Hand Surg.

[7] Bulstrode NW, Jemec B, Smith PJ (2005). The complications of Dupuytren's contracture surgery. J Hand Surg.

